# Clinical use of platelet-rich fibrin in the repair of non-healing incision wounds after fibular fracture surgery: A case report

**DOI:** 10.1097/MD.0000000000027994

**Published:** 2021-12-17

**Authors:** Xingzhen Lin, Manhua Zhu, Juan Yuan, Fang Zhi, XinJu Hou

**Affiliations:** Nanchang Hongdu Hospital of Traditional Chinese Medicine, Nanchang, Jiangxi, Province, China.

**Keywords:** platelet rich fibrin, postoperative distal tibial open fracture, skin nonunion

## Abstract

**Rationale:**

This report describes rehabilitation for a 53-year-old female recovering from non-healing skin and soft tissue defect after distal tibial open fracture by using platelet-rich fibrin topical repair following an automobile accident.

**Patient concerns:**

The patient was admitted to a rehabilitation specialty hospital approximately 1 year post a fracture of the distal left tibiofibula and separate surgical tibiofibular fracture incision and internal fixation + bone grafting.

**Diagnoses:**

Clinical presentation included the left ankle incision was interrupted for about 3 cm with poor healing, a small amount of muscle necrosis, fat liquefaction, a large amount of yellow purulent secretion overflow and necrotic material was seen in the local wound.

**Interventions:**

Platelet rich fibrin (PRF) gel was injected into the wounds and submerged sites to make full contact with the wounds and close the wounds with the autologous platelet-rich fibrin prepared by mixing, and then covered with oil gauze to keep the wounds moist and promote granulation growth. The outermost layer was covered with cotton pads.

**Outcomes:**

After 30 days of 2 PRF treatments, the skin defect was healed and no significant abnormality was observed at 6 months follow-up.

**Lessons:**

Treatment with topical autologous platelet-rich plasma gel Significantly accelerates the healing of wounds, shortens healing time, improves healing quality and reduces scar formation without significant adverse effects.

## 1 Introduction

Tibiofibular fractures are more common among limb fractures, and due to local anatomical characteristics, they are prone to fracture opening and combined with skin defects after trauma, and the treatment is more difficult due to poor local blood flow and tissue coverage.^[1,2]^ If the trauma is not properly treated at an early stage, it will lead to infection of the trauma surface, which will make the treatment more difficult. At the same time, if not properly treated, it will lead to bone infection, non-healing or delayed healing of the fracture, which will bring physiological and psychological pain to the patient in all aspects.

Recent studies have shown that platelet rich fibrin (PRF) plays an important and complex role in the repair phase of various traumatic injuries.^[3]^ PRF contains high concentrations of platelets, all the coagulation factors and many other factors that promote tissue repair (PDGF, VEGF, IGF, TGF, EGF, etc. have been detected).^[4]^ Autologous platelet-rich plasma has a unique advantage in promoting tissue repair and regeneration. On the one hand, PRF-rich factors play an important role in cell proliferation and differentiation.^[5]^ On the other hand, PRF contains a large amount of fibrin, fibronectin and hyaluronan, and the fibrin scaffold formed after coagulation is useful for promoting cell adhesion and preventing cell loss.^[6]^ At the same time, the high leukocyte content of PRP gel also inhibits the growth of many bacteria.^[7]^

This paper reports a case of non-healing skin soft tissue defect after distal tibial open fracture repair using platelet-rich plasma topical coverage with good therapeutic results, which is reported as follows.

## 2 Case presentation

### 2.1 Clinical history

The patient, a 53-year-old female, was admitted to the hospital on April 05, 2019 in a car accident resulting in a fracture of the distal left tibiofibula, and was given routine heel traction and symptomatic treatment to reduce swelling after admission. On April 18, 2019, we performed “tibiofibular fracture incision and internal fixation + bone grafting,” after which we were given symptomatic treatment for swelling and pain relief. He was discharged from the hospital on May 16, 2019, and at the time of discharge there was still about 3 cm of poorly healed skin and localized redness and swelling in the lower and middle sections of the left ankle incision, and then continued to have the left ankle dressing changed at the local community hospital, and the wound never healed. One year after the operation, he was readmitted to the hospital for rehabilitation.

### 2.2 Assessment

**Figure 1:**
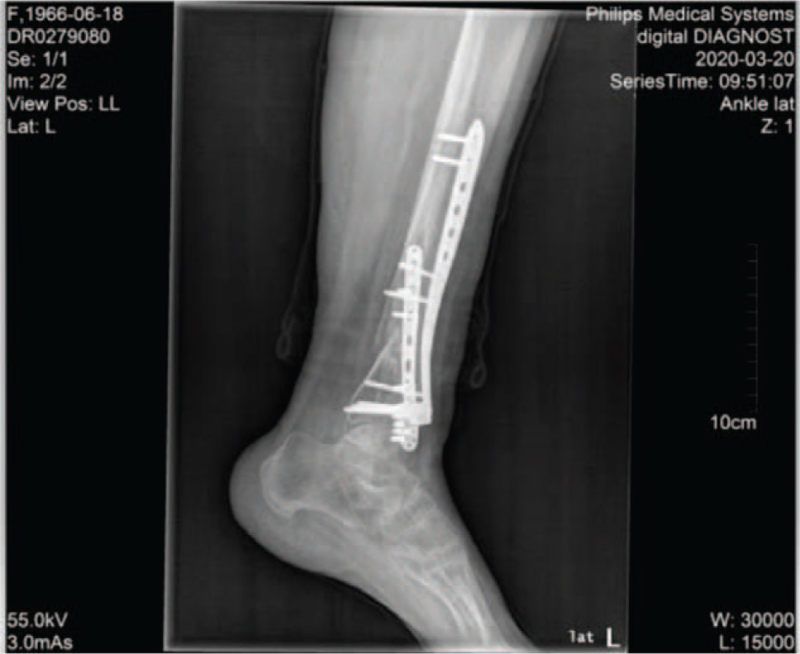
X-ray of fracture of the distal left tibiofibula.

A follow-up X-ray showed that the metal internal fixation of the left inferior tibiofibular fracture was in place, no loosening was seen, the alignment of the severed end was acceptable, and the left inferior tibial segment was mildly osseointegrated (Fig. 1).

On examination: the left ankle incision was interrupted for about 3 cm with poor healing, a small amount of muscle necrosis, fat liquefaction, a large amount of yellow purulent secretion overflow and necrotic material was seen in the local wound. The wound did not improve after routine dressing changes.

### 2.3 Platelet-rich fibrin production

**Figure 2:**
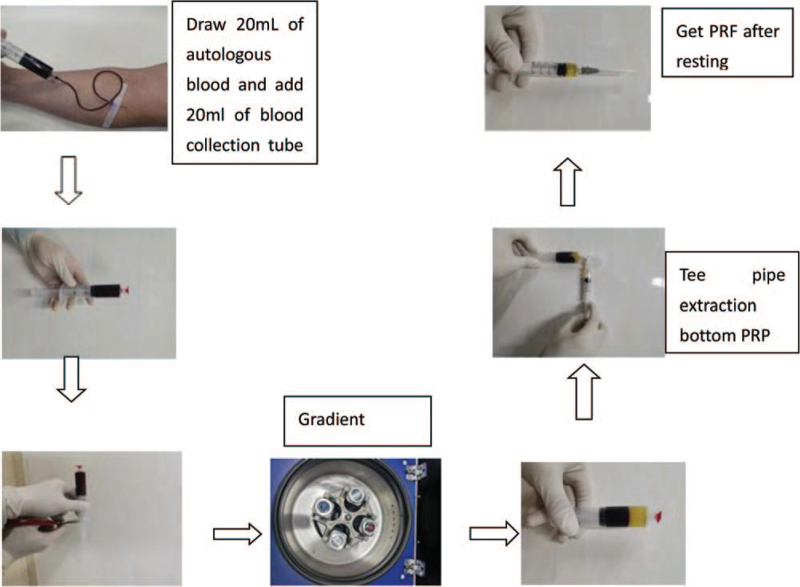
Platelet-rich fibrin production.

PRF gel preparation and application the preparation process was strictly aseptic: - A disposable screw-in 20 mL syringe with an 18G needle was used to draw 20 mL of autologous blood under strict aseptic conditions, and the screw-in syringe was tightened with a disposable plug, and the posterior projection and tail were cut off. Weigh, level, and centrifuge at 3000 r/min for 10 minutes. After resting, the blood sample was divided into 3 layers, the upper layer of yellowish clarified liquid was platelet plasma, the lower layer of red loose jelly-like material was red cell debris, and the middle layer of yellowish gel was PRF gel, and the PRF gel was obtained by removing the PRF gel using a three-way connecting tube, and then it was rested in a dry sterilized container for 10 minutes to obtain the PRF gel (Fig. 2).

### 2.4 Treatment

**Figure 3:**
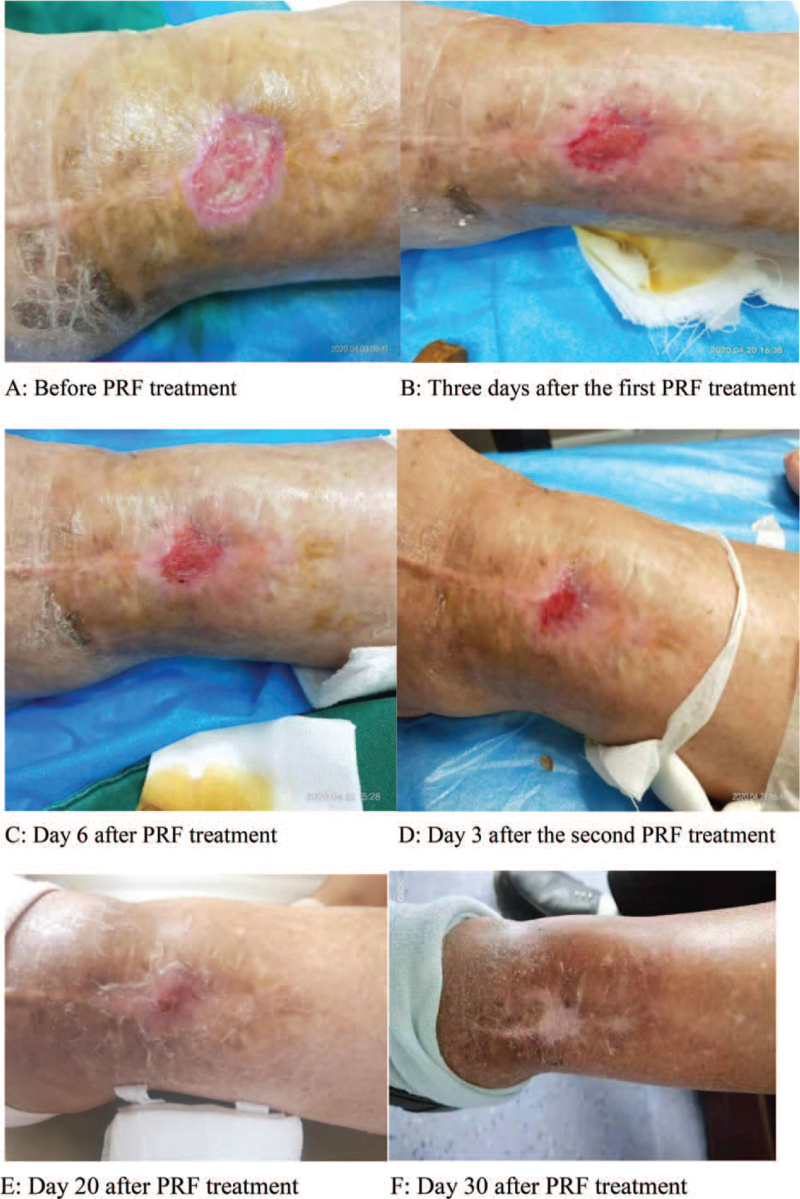
Comparison of the patient's effect before and after treatment of the skin lesion on the anterior margin of the left tibia.

Admission to the hospital to improve blood routine, blood sedimentation, C-reactive protein and other tests to exclude contraindications. Necrotic tissue was scraped out of the wound with a sterile blade (see extensive muscle necrosis, fat liquefaction, and large amounts of purulent secretions overflowing as in Fig. 3A) until the wound oozed blood. Then, after rinsing with saline, PRF was injected into the wound and the submerged area, so that the autologous platelet-rich fibrin mixed with the preparation could fully contact with the wound and close the wound, and then covered with oil gauze to keep the wound moist and promote the growth of granulation. The wound was covered locally with PRF on April 17, 2020, bandaged and covered with PRF again after 1-week interval.

## 3 Results

The size of the wound was measured using the Image J tool. The size of the wound before PRF treatment was 2.90∗2.80 cm and a small amount of muscle necrosis, fat liquefaction, spillage of yellow purulent discharge and necrotic material was visible in the local wound (Fig. 3A); Three days after the first PRF treatment, the size of the wound was 2.04∗2.07 cm, the yellow pus around the wound disappeared and a small amount of necrotic material and a large amount of bright red granulation tissue was visible (Fig. 3B). On the sixth day after PRF treatment, the wound area was reduced to 1.38∗1.53 cm in size, the local wound was dry, the exudate disappeared and the granulation tissue was bright red (Fig. 3C). On the 3rd day after the second PRF treatment, the wound was further reduced to 0.48∗0.72 cm after routine dressing change, the local wound was dry, the exudate disappeared and the granulation tissue was bright red (Fig. 3D). On the 20th day after treatment, the local wound was dry and healed with a local crust (Fig. 3E). After the 30th day of treatment, the wound disappears and the local wound heals fibrously (Fig. 3F).

## 4 Discussion

Tibiofibular fractures are mostly due to direct violence and are a common clinical fracture condition. Because the anterior medial tibia is located under the skin and has angles, the fracture segment breaks under external forces are very likely to penetrate the skin and form an open fracture, and the lower 1/3 of the tibiofibular fracture, often due to poor blood flow and little tissue coverage, and the characteristic wound of the built-in object often further slows tissue repair, resulting in delayed healing or non-healing of the fracture, along with non-healing of the surrounding skin soft tissue defect.^[8]^ Current research suggests that skin tissue repair and healing is a complex and orderly biological process of regeneration, repair and reconstruction, showing a high degree of integrity and network. Under the regulation of the body, inflammatory cells, repair cells, extracellular matrix and cytokines are coordinated to participate in wound healing.^[9]^ platelets play an important role in the process of angiogenesis, tissue repair and inflammatory response. It is generally believed that there are 4 main causes of non-healing skin tissue defects:

1. the presence of wound infection or necrotic tissue;
2. impaired microcirculation of wound blood supply;
3. reduced number and activity of local growth factors or uncontrolled regulation of multiple growth factor networks;
4. altered scaffolding and excessive apoptosis of repair cells and structural changes of receptors on cell membranes, resulting in loss of coupling between growth factors and receptors.^[10]^

Therefore, the supplementation of fresh trauma repair cells and active growth factors has become a key factor in the repair of skin soft tissue defects after tibiofibular fracture surgery at present. PRF is an autologous platelet concentrate rich in cytokines and growth factors that has a molecular structure similar to that of a natural blood clot and provides a site for migration, proliferation and differentiation of tissue cells. Current studies have demonstrated that PRF contains a variety of growth factors that act synergistically to promote the synthesis of type I collagen and fibronectin, promote the chemotaxis and proliferation of stromal stem cells, and stimulate the differentiation and proliferation of fibroblasts and vascular endothelium, which play an important regulatory role in the process of tissue healing.^[11]^ The three-dimensional mesh structure of PRF as a matrix provides a favorable place for cell attachment, migration and differentiation, enabling tissue cells and stem cells from circulating blood to grow into it faster;^[12]^ A large number of lagging platelets and growth factors are chemically bonded to fibrin, and this special structure has a strong affinity for a variety of growth factors, which results in the slow release of growth factors and prolongs the action time of PRF in the wounds;^[13]^ It has also been shown that the three-dimensional structure of PRF can trap most leukocytes, modulate the inflammatory response, and cause a slow and sustained release of multiple cytokines, resulting in a positive effect on tissue repair.^[14]^ Crisci^[15]^ used L-PRF for the treatment of diabetic ulcers and found good results when L-PRF gel was placed on the wound after surgical debridement. Yu^[16]^ found that L-PRF film induced wound healing in an adult male rat pressure ulcer model and suggested that it may be a natural and effective tool for wound healing.

Combined with the treatment procedure in this case, it can be concluded that the important role of PRF in promoting wound healing in skin tissue was initially verified in this case. The application of PRF to non-healing wounds with soft tissue defects can effectively accelerate repair, and PRF is obtained from autologous blood, easy to obtain and convenient to operate, so PRF has great research potential and practical value for the repair of skin and soft tissue defects after tibiofibular fracture surgery.

## Author contributions

**Conceptualization:** Xingzhen Lin, Xinju Hou.

**Data curation:** Xingzhen Lin, Manhua Zhu.

**Formal analysis:** Juan Yuan, Fang Zhi.

**Investigation:** Xingzhen Lin.

**Methodology:** Xingzhen Lin, Xinju Hou.

**Validation:** Xinju Hou.

**Writing – original draft:** Xingzhen Lin, Xinju Hou.

**Writing – review & editing:** Xingzhen Lin, Xinju Hou.
